# Targeting Purinergic Receptor P2RX1 Modulates Intestinal Microbiota and Alleviates Inflammation in Colitis

**DOI:** 10.3389/fimmu.2021.696766

**Published:** 2021-07-20

**Authors:** Xu Wang, Xiao Yuan, Yuting Su, Jing Hu, Qian Ji, Shengqiao Fu, Rongkun Li, Lipeng Hu, Chunhua Dai

**Affiliations:** ^1^ Department of Radiation Oncology, Institute of Oncology, Affiliated Hospital of Jiangsu University, Zhenjiang, China; ^2^ State Key Laboratory of Oncogenes and Related Genes, Shanghai Cancer Institute, Shanghai Jiao Tong University, Shanghai, China

**Keywords:** purinergic signaling, P2RX1, inflammation, inflammatory bowel disease (IBD), purinergic receptor

## Abstract

Inflammatory bowel disease (IBD) remains one of the most prevalent gastrointestinal diseases worldwide. Purinergic signaling has emerged as a promising therapeutic target of inflammation-associated diseases. However, little is known about the specific roles of purinergic receptors in IBD. In the present study, expression profile of purinergic receptors was screened in the public Gene Expression Omnibus (GEO) datasets, and we found that expression of P2RX1 was significantly upregulated in inflamed colon tissues. Then, purinergic receptor P2RX1 was genetically ablated in the background of C57BL/6 mice, and dextran sulfate sodium (DSS) was used to induce mice colitis. RNA sequencing results of colon tissues showed that genetic knockout of P2RX1 suppressed the inflammation responses in DSS-induced mice colitis. Flow cytometry indicated that neutrophil infiltration was inhibited in P2RX1 ablated mice. 16S ribosomal DNA sequencing revealed major differences of intestinal microbiota between WT and P2RX1 ablated mice. Functional metagenomics prediction indicated that the indole alkaloid biogenesis pathway was upregulated in *P2RX1* gene ablated mice. Further studies revealed that microbiota metabolites (indole alkaloid)-involved aryl hydrocarbon receptor (AhR)/IL-22 axis was associated with the beneficial effects of P2RX1 ablation. Finally, we found that a specific P2RX1 inhibitor succeeded to improve the therapeutic efficiency of anti-TNF-α therapy in DSS-induced mice colitis. Therefore, our study suggests that targeting purinergic receptor P2RX1 may provide novel therapeutic strategy for IBD.

## Background

Inflammatory bowel disease (IBD), which includes Crohn’s disease (CD) and ulcerative colitis (UC), that affects the whole gastrointestinal tract and the colon only, remains one of the most prevalent gastrointestinal diseases worldwide (1). Although the precise etiology of IBD is not fully understood, many factors have been found to contribute to the progress of IBD, including genetic susceptibility, host immune system dysregulation, and environmental factors such as intestinal microbial flora (2, 3). Anti-tumor necrosis factor-α (anti-TNF) therapy has greatly improved the clinical outcome of IBD (4). However, despite their efficacy, one third of the patients receiving anti-TNF-α agents are primary non-responders and nearly half of the patients that initially respond may subsequently lose response (5). According to previous reports, the effectiveness of a specific anti-TNF-α antibody in CD varied between 37 and 64% for response, and 24 and 42% for remission at week 14, respectively; for UC, the rates of response and remission were 37–57% and 24–26%, respectively (6). Improving the efficacy of anti-TNF therapy may facilitate the clinical prognosis of IBD (7).

Adenosine triphosphate (ATP) is an important energy currency inside the cell. However, under inflammatory conditions, abundant ATP is secreted from activated cells *via* ATP-releasing channels, or directly liberated from apoptotic or necrotic cells (8). By activating various purinergic receptors in the gastrointestinal system, extracellular ATP has recently been linked with the progression of IBD (9). The purinergic receptor P2RX1 belongs to a family of ATP-gated ion channels, which comprise seven receptor subunits (P2RX1–7) that assemble to form a variety of homotrimeric and heterotrimeric receptors (8). Once activated by extracellular ATP under stress or inflammatory conditions, P2RX1 can induce fast Ca^2+^ influx, which subsequently strengthens the activation of cells. Our group and others have highlighted the role of P2RX1 in inflammatory activation of immune cells (10, 11); however, the specific involvement of P2RX1 in IBD remains largely unknown.

Aryl hydrocarbon receptor (AhR) is an important cytosolic ligand-activated transcription factor. Accumulating evidence has proved that activation of AhR by endogenous metabolites can modulate various essential biological processes such as inflammation regulation, cell differentiation, and tissue repair (12). With respect to IBD, gut microbiota-mediated metabolism of tryptophan into indole derivatives provide the ligands for AhR, which subsequently drives IL-22-dependent regeneration of mucosal barrier integrity and reduces pro-inflammatory potential (13). Manipulation of gut microbiota to produce indole derivatives is promising for the treatment of IBD.

To explore the potential effects of extracellular ATP on colitis, we screened all the purinergic receptors in both human and mouse transcriptional datasets and found that P2RX1 was significantly upregulated in inflamed colon and associated with anti-TNF-α therapy efficiency. Using P2RX1 knockout mice, we observed that P2RX1 ablation significantly relieved dextran sulfate sodium (DSS)-induced mouse colitis. In addition, combination of a P2RX1 inhibitor facilitated the therapeutic efficiency of TNF-α monoclonal antibody (mAb) in mice colitis.

## Materials and Methods

### Animal Models

All animal experiments were undertaken in accordance with the National Institutes of Health Guide for the Care and Use of Laboratory Animals. With the help from GemPharmatech Co., Ltd., (Jiangsu, China) *P2rx1* ^−/−^ mice were constructed in the C57BL/6 background with CRISPR/Cas9 system. Guide RNA sequences targeting Exon2–Exon3 of P2rx1 were: S1: CATCCAACGACGCAAGTGGC (PAM: TGG); S2: TGGTTCGCAAGTAGTTTCCC (PAM: AGG); S3: ATGGTGCTGTTGCGGGGCAC (PAM: TGG); S4: TTGGTCCTTGGTGGTAATGT (PAM: GGG). No obvious differences of selective fertilization/mortality *in utero*, development, and growth were observed among *P2rx1* ^−/−^, heterozygous, and WT littermates. Nevertheless, we found that genetic ablation of P2RX1 resulted in male infertility, which was consistent with a previous report indicating that P2RX1 was involved in neurogenic vas deferens contraction (14). Homozygous *P2rx1* ^−/−^ mice and WT littermates were always generated from heterozygous parents. Following primers were used for genotyping WT, *P2rx1* ^−/−^ and heterozygous mice: forward: CACAGCCTTTGCTAGTGCCA, reverse: AGTGCAGCCACTGTCATCTT (**Supplementary Figure 7**). *P2rx1* ^−/−^ mice and WT littermates were cohoused after weaning and during the experiment in a specific pathogen-free facility under a 12-h light/dark cycle with free access to food and water. To induce colitis, 6–8 weeks WT or *P2rx1* ^−/−^ mice were treated with 2 or 3% DSS [molecular weight (MW) = 36,000–50,000 Da; MP Biomedicals) dissolved in drinking water. Neutralizing anti-TNF-α mAb (0.1 mg/day, Biolegend) and P2RX1 inhibitor (NF449, 10 mg/kg/day, Tocris) were intraperitoneally injected for 7 days after DSS was administrated daily. The severity of colitis was assessed using the disease activity index (DAI) as previously described (15).

### RNA Sequencing

RNA sequencing was performed as previously reported (16). Briefly, total RNAs were isolated using the TRIzol reagent following the instructions of the manufacturer and sent to GCBI Co., Ltd. for clustering and sequencing. The library fragments were purified with AMPure XP system. The clustering of the index-coded samples was performed on a cBot Cluster Generation System using TruSeq PE Cluster Kit v3-cBot-HS (Illumia) according to the instructions of the manufacturer. After cluster generation, the library preparations were sequenced on an Illumina Hiseq X Ten and 150 bp paired-end reads were generated. HTSeq v0.6.0 was used to count the read numbers mapped to each gene. Then TPM of each gene was calculated based on the length of the gene, and read count was mapped to this gene.

### 16S Ribosomal DNA Sequencing and Analyzing

16S rDNA sequencing was performed as previously reported (15). DNA was extracted from fresh stool samples according to established protocols using a method combining mechanical disruption (bead-beating) and phenol/chloroformbased purification. The hypervariable V3–V4 regions of the bacterial 16S rRNA gene were PCR amplified by using the primer pairs 338F 5′‐ACTCCTACGGGAGGCAGCAG‐3′ and 806R 5′‐GGACTACHVGGGTWTCTAAT‐3′. PCR amplification was carried out in a 25 ml reaction system using an optimized and standardized 16S-amplicon-library preparation protocol. Purified amplicons were pooled in equimolar concentrations (11 ng DNA for each sample) and paired-end sequenced (2 × 300) on an Illumina MiSeq platform. The 16S rRNA sequencing data were processed using the Quantitative Insights Into Microbial Ecology (QIIME) (version 1.9.1) platform in an online Majorbio cloud platform. The raw sequence reads were filtrated with a minimum overlap of 10 bp and a maximum mismatch ratio 0.2 by using FLASH (version 1.2.11). Operational taxonomic units (OTUs) were picked at 97% similarity cut-off, and the identified taxonomy was then aligned using the Greengenes database (version 13.5). Chimeric sequences were identified and removed in the process of clustering with the software of USEARCH (version 7). OTUs with number of sequences < 20 of the total number of sequences were removed from the OTU table with the software of USEARCH.

Phylogenetic Investigation of Communities by Reconstruction of Unobserved States (PICRUSt) was used to perform the functional metagenomics prediction (17). In brief, PICRUSt was first used to correct biom tables for 16S rRNA copy numbers and subsequently used to predict KEGG (Kyoto Encyclopedia of Genes and Genomes) orthologs (KO). The maximum allowed Nearest Sequenced Taxon Index (NSTI) value was set to 2 to control for the overall accuracy of the metagenomic predictions. The gene function predicts the spectrum, and finally the sequence composition obtained by sequencing is matched with the database to predict the metabolic function of the flora.

### Inflammatory Cytokines Detection

RT-qPCR was performed to detect the mRNA expression of inflammatory cytokines. Total RNAs were isolated using the TRIzol reagent following the instructions of the manufacturer. Real-time PCR analyses were applied for gene expression study, and SYBR Premix Ex Taq (Roche) was used to run PCR on a 7500 Real-time PCR system (Applied Biosystems) at the recommended thermal settings. Relative mRNA expression was calculated using the 2^(−ΔΔCt)^ method and normalized to *β*-actin mRNA levels. The following primers were used: IL-1β-F: CACGATGCACCTGTACGATCA, IL-1β-R: GTTGCTCCATATCCTGTCCCT; IL-6-F: AGTTGCCTTCTTGGGACTGA, IL-6-R: TCCACGATTTCCCAGAGAAC; TNF-α-F: GACGTGGAACTGGCAGAAGAG, TNF-α-R: TTGGTGGTTTGTGAGTGTGAG; Cxcl1-F: TCTCCGTTACTTGGGGACAC, Cxcl1-R: CCACACTCAAGAATGGTCGC-3; IL-22-F: CATGCAGGAGGTGGTACCTT, L-22-R: CAGACGCAAGCATTTCT. IL-22 protein was detected by a commercialized ELISA kit (Abcam) according to the instructions of the manufacturer.

### Flow Cytometry

Lamina propria (LP) mononuclear cells were obtained from colonic specimens as previously reported (18). Briefly, mouse intestine was harvested, and then fat tissues were removed. The colon was opened longitudinally and cut into pieces. The colons were then washed three times with PBS containing 2 mM EDTA, and supernatants were collected and passed through a 70 μm cell strainer. The remaining LPs were collected and digested with collagenase and DNase. LP immune cells were separated by collecting the interface fractions between 40 and 80% Percoll. anti-Ly6G-APC (Biolegend, 1A8, 1:100) antibody was used for staining neutrophils, and anti-F4/80-FITC (Biolegend, BM8, 1:100) antibody was used for staining macrophages. For P2RX1 staining, anti-P2RX1 (Alomone, APR-022, 1:80) antibody followed by an anti-rabbit-PE secondary antibody (Abcam, 1:200) was used. All flow cytometry was performed on Aria II (BD).

### High-Performance Liquid Chromatography Analysis

Thawed stools (100 mg) were mixed with 700 ml cold 50% methanol. The mixture was homogenized and centrifuged at 12,000 g for 10 min and supernatant was transferred to new tubes, mixed with 600 ml cold 50% methanol, and then centrifuged at 12,000 g for 10 min. The supernatant fluid was mixed with equal volume of working solution (0.12% benzoic acid:50% methanol = 1:1). The mixture was mixed thoroughly and centrifuged at 12,000 g for 10 min. After filtering through a 0.45 mm filter, the IAA and indole in the supernatant were detected.

### Statistical Analysis

Data are presented as mean ± standard deviation. Reproducibility was ensured by performing more than three independent experiments. All statistics were carried out using GraphPad Prism 7.0. Student’s t-test was used to compare the differences between two groups. One-way analysis of variance (ANOVA) and Tukey’s test were used for the three or more group comparisons. Survival was analyzed with log-rank test. A value of *P <*0.05 was considered to be statistically significant.

## Results

### Purinergic Receptor P2RX1 Expression Is Increased in Activated Colitis

**Figure 1:**
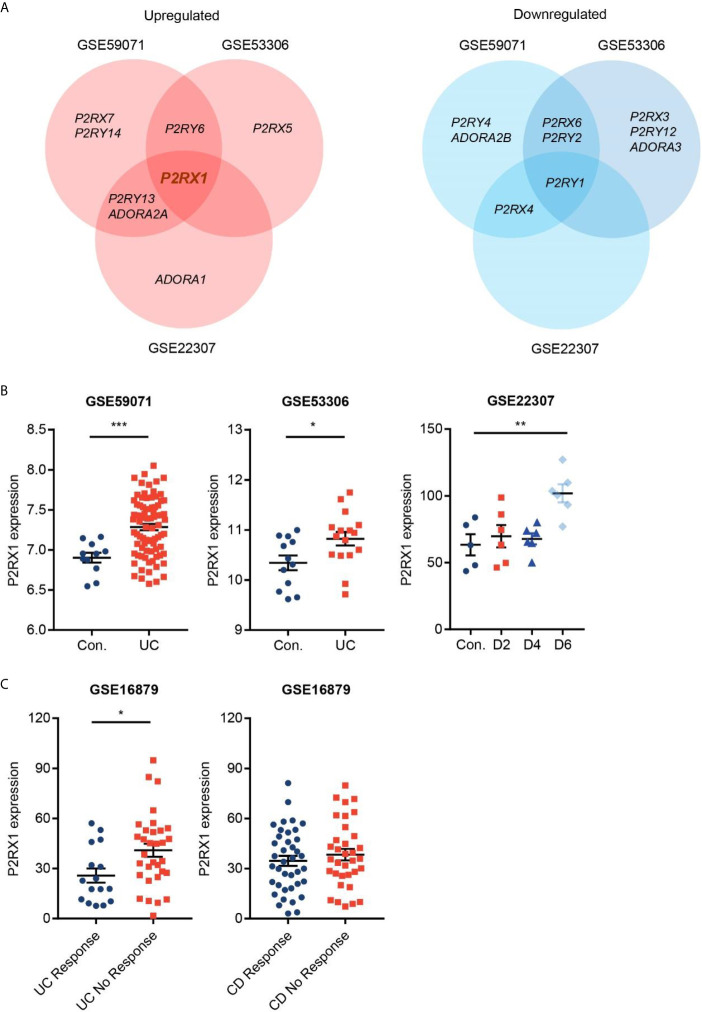
Purinergic receptor P2RX1 expression is increased in activated colitis. **(A)** Expression of purinergic receptors were analyzed in two Gene Expression Omnibus (GEO) datasets (GSE59071 and GSE53306) containing expression profile of actively inflamed mucosa from colitis patients, and one GEO dataset (GSE22307) containing expression profile of colon tissues from DSS-induced mouse colitis. Venn diagram of significantly upregulated and downregulated genes was shown. **(B, C)** Expression profiles of P2RX1 in the GSE59071, GSE53306, GSE22307, and GSE16879 datasets. *P < 0.05, **P < 0.01, and ***P < 0.001.

To identify the potential purinergic receptors that were dysregulated in colitis, we first analyzed two Gene Expression Omnibus (GEO) datasets (GSE59071 and GSE53306) containing expression profile of actively inflamed mucosa from colitis patients, and one GEO dataset (GSE22307) containing expression profile of colon tissues from DSS-induced mouse colitis. The samples from the two clinical datasets were obtained from UC patients, and the samples from murine colitis was induced by DSS which resembled the features of human UC (19). By screening seven purinergic P2X receptors (P2RX1–7), eight P2Y receptors (P2RY1/2/4/6/11/12/13/14), and four P1 receptors (ADORA1/A2A/A2B/A3R), we found that expression profiles of purinergic receptors were substantially reprogrammed (**Figure 1A**). Eight receptors, including P2RX7, P2RY6 and P2RY5, were significantly upregulated, whereas nine receptors, including P2RY4, ADORA2B and P2RX6, were significantly downregulated. Venn diagram indicated that P2RX1 was overlapped in the intersection among the upregulated purinergic receptors (**Figures 1A, B** **)**, suggesting that P2RX1 is of potential importance to colitis. Anti-tumor necrosis factor (TNF) therapy has greatly improved the medical treatment of inflammatory bowel diseases, and infliximab was the first approved anti-TNF agent (20). Nevertheless, up to 30% of patients show no clinical benefit following infliximab treatment, and up to 50% lose response over time. Notably, in another GEO dataset (GSE16879) containing results of clinical responses to infliximab based on endoscopic and histologic findings, we noticed that P2RX1 expression was significantly higher in infliximab-no-response UC patients than that in infliximab-response UC patients (**Figure 1C**). However, in CD patients, there were no significant differences between infliximab-no-response and infliximab-response patients (**Figure 1C**), which further depicted the important involvement of P2RX1 in UC.

### P2RX1 Ablation Relieves DSS-Induced Mouse Colitis

**Figure 2:**
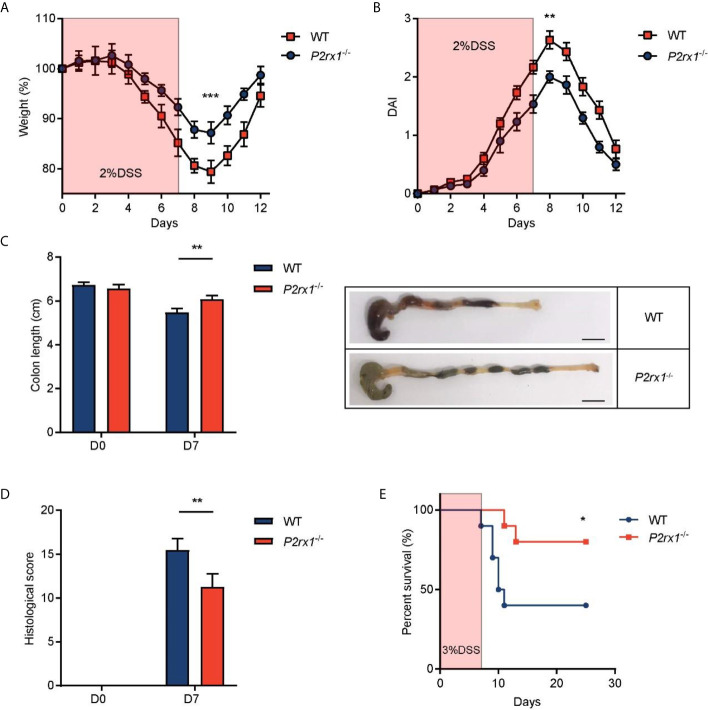
P2RX1 ablation relieves DSS-induced mouse colitis. **(A, B)** WT and *P2rx1* ^−/−^ mice were treated with 2% DSS for 7 days. Weight loss **(A)** and disease activity index (DAI) **(B)** were calculated (n = 6 per group). **(C)** Colon length of WT and *P2rx1* ^−/−^ mice was measured at days 0 and 7. Representative colon tissues at day 7 were shown (n = 6 per group). Scale bar is 1 cm. **(D)** Histologic score of WT and *P2rx1* ^−/−^ mice at days 0 and 7 was determined according to pathological examinations (n = 6 per group). **(E)** WT and *P2rx1* ^−/−^ mice were exposed to 3% DSS for 7 days, and survival status was monitored for 25 days (n = 10 per group). *P < 0.05, **P < 0.01, and ***P < 0.001.

To further study the involvement of P2RX1 in IBD, we generated P2RX1 knockout (*P2rx1* ^−/−^) mice. *P2rx1* ^−/−^ mice and control wild type (WT) littermates were challenged with 2% DSS for 7 days to induce acute experimental colitis. We observed that all of the DSS-treated mice developed colitis, whereas body weight loss and disease activity index (DAI) score were significantly lower in *P2rx1* ^−/−^ mice at the inflammatory phase (day 7) of colitis (**Figures 2A, B** **)**. *P2rx1* ^−/−^ mice displayed less severe colon shortening than WT mice (**Figure 2C**). Pathological examination showed that prior to the induction of DSS colitis, no histological changes were observed in all mice lines (**Figure 2D** and **Supplementary Figure 1**). However, during colitis, WT mice displayed more severe ulceration, extensive epithelium erosion, and inflammation cell infiltration (**Figure 2D** and **Supplementary Figure 1**). In another experiment, *P2rx1* ^−/−^ mice and control WT littermates were challenged with 3% DSS for 7 days, and survival was monitored for 25 days. We observed that 80% *P2rx1* ^−/−^ mice survived, whereas only 40% WT mice survived (**Figure 2E**). The death rate of *P2rx1* ^−/−^ mice was significantly decreased compared with that of WT mice. Together, these results suggest that P2RX1 ablation is sufficient to suppress DSS-induced mouse colitis.

### P2RX1 Ablation Restricts Inflammatory Responses in DSS-Induced Mouse Colitis

**Figure 3:**
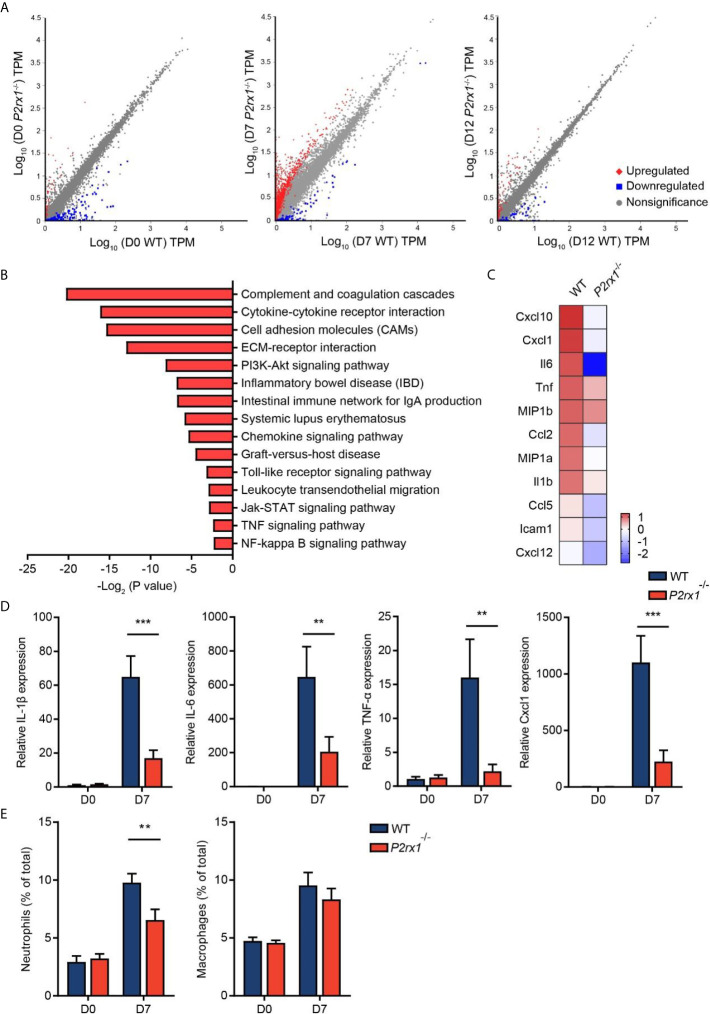
P2RX1 ablation restricts inflammatory responses in DSS-induced mouse colitis. **(A)** WT and *P2rx1* ^−/−^ mice were treated with 2% DSS for 7 days. At days 0, 7, and 12, colon tissues were harvested, and RNA sequencing was performed. Differential analysis was analyzed, with P <0.001 and fold change >4 being considered as significantly varied (n = 3 for D0 *P2rx1* ^−/−^ group, and n = 1 for the rest of the groups). **(B)** KEGG pathway analysis was performed to compare the functional enrichment genes of WT and *P2rx1* ^−/−^ mice at day 7. **(C)** A heatmap of inflammation-associated genes was shown. **(D)** Inflammation-associated genes were detected by RT-qPCR (n = 4 per group). **(E)** Neutrophils and macrophages were analyzed by flow cytometry (n = 4 per group). **P < 0.01 and ***P < 0.001.

RNA sequencing of colon tissues was performed to compare the transcriptional variations between *P2rx1* ^−/−^ and WT mice. Differential analyses revealed substantial differences of transcriptional profiles at the inflammatory phase of colitis (day 7), with 1,515 genes significantly altered (**Figure 3A**). In comparison, 162 genes and 270 genes were significantly altered at the basal phase (day 0) and recovery phase (day 12). To further identify the specific molecular events that might be involved in P2RX1 ablation at day 7, KEGG pathway analysis was performed. We found that inflammation-associated pathways were thoroughly downregulated in *P2rx1* ^−/−^ mice, including cytokine–cytokine receptor interaction, cell adhesion molecules and inflammatory bowel disease (**Figure 3B**). In addition, both RNA sequencing and RT-qPCR results showed inflammatory cytokines were significantly reduced at day 7 of *P2rx1* ^−/−^ mice (**Figures 3C, D** **)**. These results suggest that P2RX1 ablation inhibits inflammatory responses in DSS-induced mouse colitis.

Given that neutrophils and macrophages are closely related to the inflammatory process in IBD (21), and P2RX1 has been reported to participate in the inflammatory activation of neutrophils and macrophages (10, 22), we next sought to investigate the involvement of P2RX1 in inflammatory responses in DSS-induced mouse colitis. By scanning the P2RX1 expression pattern of intestinal tissue in public signal cell sequencing database (www.proteinatlas.org), we found that P2RX1 was not expressed in enterocytes or Paneth cells (**Supplementary Figure 2**). Instead, it was noticed that P2RX1 was highly expressed in granulocytes (**Supplementary Figure 2**). The public database did not show the expression pattern of P2RX1 in intestinal macrophages. Next, flow cytometry was performed to determine the involvement of P2RX1 in neutrophil and macrophage infiltration in colitis. As previously reported (23), our results showed that DSS could significantly increase the infiltration of neutrophils and macrophages (**Figure 3E** and **Supplementary Figures 3A, B** **)**. Interestingly, we noticed that neutrophil infiltration was reduced in *P2rx1* ^−/−^ mice when compared with WT mice (**Figure 3E** and **Supplementary Figures 3A**). However, macrophage infiltration was not changed (**Figure 3E** and **Supplementary Figures 3B**). This result might be explained by that P2RX1 expression was significantly higher in neutrophils than in macrophages (**Supplementary Figures 3A, B** **)**.

### Intestinal Microbiota Is Altered in DSS-Treated *P2rx1* ^−/−^ Mice

**Figure 4:**
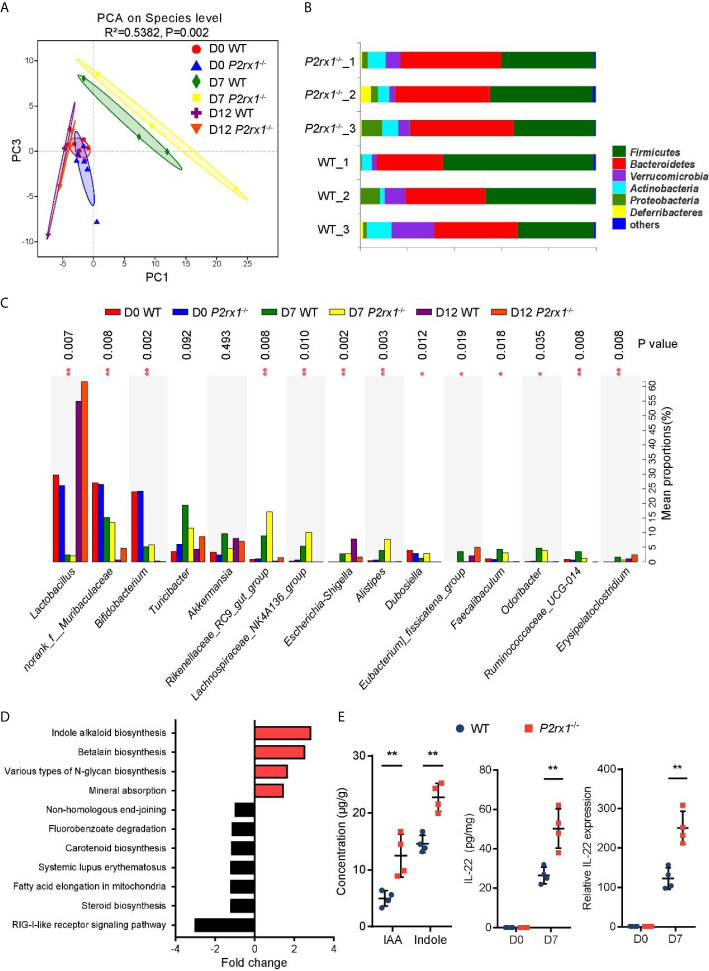
Intestinal microbiota is altered in DSS-treated *P2rx1* ^−/−^ mice. **(A)** WT and *P2rx1* ^−/−^ mice were treated with 2% DSS for 7 days. At days 0, 7 and 12, fecal microbiota was quantified using 16S rDNA sequencing. Principal component analysis (PCA) was performed based on the operational taxonomic units (OTUs) composition (n = 6 for D0 WT and *P2rx1* ^−/−^ groups, n = 3 for the rest groups). **(B)** Relative abundance of bacterial phylum in the fecal microbiota was analyzed. **(C)** Detailed comparison of bacterial genus in WT and *P2rx1* ^−/−^ mice was performed. **(D)** Functional metagenomics prediction analysis based on the result of 16S rDNA gene sequencing using PICRUSt1 was performed. **(E)** Fecal indole-3-acetic acid (IAA) and indole at day 7 were detected. IL-22 mRNA and protein at day 0 and day 7 were determined (n = 4 per group). **P < 0.01.

Accumulating evidence has identified that specific intestinal microbiota or microbial products may regulate the outcome of intestinal inflammation (24). To determine whether the suppressed inflammation in DSS-induced *P2rx1* ^−/−^ mice was associated with intestinal microbiota, 16S rDNA sequencing was performed to clarify the microbial composition. Principal component analysis (PCA) revealed that the operational taxonomic units (OTUs) of the microbial communities were substantially modulated at days 0, 7, and 12 after DSS-treatment, and major differences between WT and *P2rx1* ^−/−^ mice at day 7 were observed (**Figure 4A**). At phylum level, composition of *Firmicutes* was minimally reduced, whereas *Bacteroidetes* were significantly increased in *P2rx1* ^−/−^ mice as compared to WT mice (**Figure 4B**). At genus level, DSS-treatment decreased the probiotics *Lactobacillus* and *Bifidobacterium* and increased the pathogenic bacteria *Escherichia*–*Shigella* (**Figure 4C**). The detailed composition of species was shown in **Supplementary Figure 4**. To further investigate the specific contribution of intestinal microbiota to the host inflammation, functional metagenomics prediction of a total 328 KEGG pathways was analyzed, including metabolism, cellular processes, environmental information processing, *etc.* We found that Indole alkaloid biogenesis pathway was the most upregulated in the intestinal microbiota of *P2rx1* ^−/−^ mice (**Figure 4D**). It does not exceed our expectation because *Lactobacillus* has been reported to possess the ability to convert tryptophan to indole alkaloids and active aryl hydrocarbon receptor (AhR) to alleviate murine colitis (15). Moreover, previous reports indicate that indole can be produced from tryptophan through the gut microbiota that mainly is distributed within *Clostridiaceae*, *Bacteroidaceae*, *Rikenellaceae*, *Lachnospiraceae*, and *Enterobacteriaceae* families (25). Our results showed that *Clostridiaceae*, *Bacteroidaceae*, *Rikenellaceae*, and *Lachnospiraceae* families were all enriched in *P2rx1* ^−/−^ mice at day 7, except for the *Enterobacteriaceae* family (**Supplementary Figure 5**). It further indicated that these bacteria might enhance the indole alkaloids production.

Indole alkaloids have been proved as essential protective agents in colitis by activating AhR and inducing subsequent IL-22-depedent regeneration of mucosal barrier integrity (26, 27). Next, indole and an important class of indole alkaloid, indole-3-acetic acid (IAA), were detected. Results showed that indole and IAA were significantly increased in DSS-exposed *P2rx1* ^−/−^ mice at day 7 (**Figure 4E**). In addition, we found that IL-22 mRNA and protein levels were both upregulated in *P2rx1* ^−/−^ mice (**Figure 4E**). *CYP1A1* and *CYP1B1*, two target genes of AhR, were also increased in *P2rx1* ^−/−^ mice (**Supplementary Figure 6**). It suggests that the beneficial effect of P2RX1 deficiency in IBD is associated with microbiota metabolite-involved AhR/IL-22 axis.

### Inhibition of P2RX1 Promotes the Efficiency of Anti-TNF-α Therapy in Mouse Colitis

**Figure 5:**
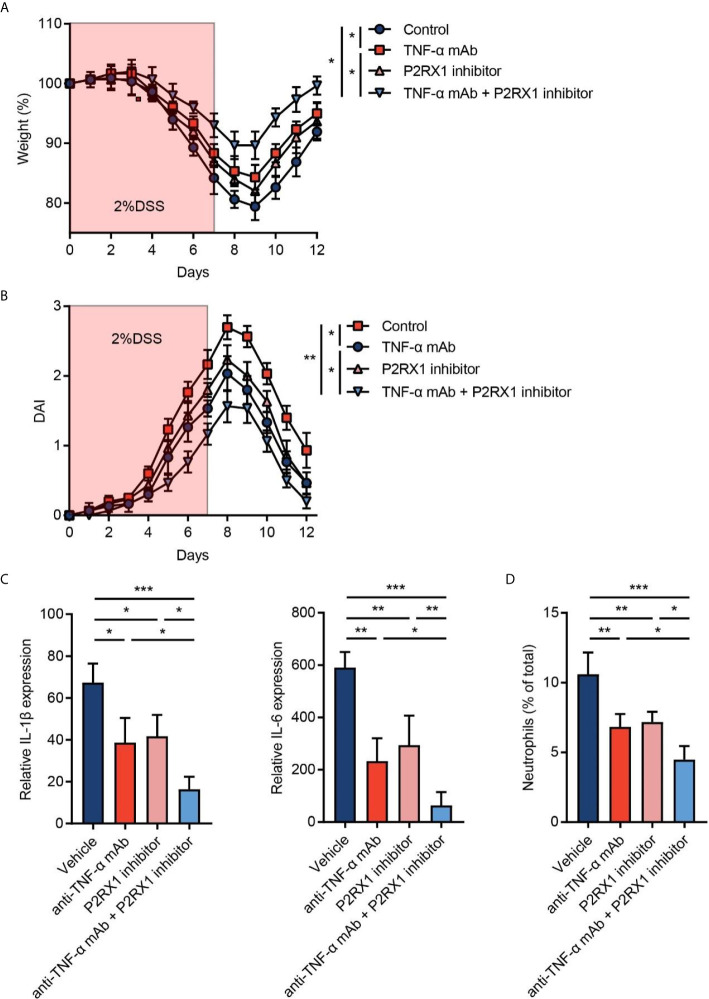
Inhibition of P2RX1 promotes the efficiency of anti-TNF-α therapy in mouse colitis. **(A, B)** WT mice were treated with 2% DSS for 7 days. Anti-TNF-α monoclonal antibody (mAb) (0.1 mg/day) or/and P2RX1 inhibitor (NF449, 10 mg/kg/day) was/were administrated for 7 days. Weight loss **(A)** and disease activity index (DAI) **(B)** were calculated (n = 6 per group). Statistical analyses were performed at day 7. **(C)** At day 7, RT-qPCR was performed to detect inflammatory cytokine expression (n = 4 per group). **(D)** At day 7, colonic neutrophils were determined by cytometry (n = 4 per group). *P < 0.05, **P < 0.01, and ***P < 0.001.

Given that P2RX1 expression showed a correlation with anti-TNF-α responses in clinical UC patients (**Figure 1C**), we speculated whether combination of P2RX1 inhibitor and TNF-α monoclonal antibody (mAb) could achieve a better therapeutic efficacy than TNF-α mAb treatment alone. To address this issue, TNF-α mAb was applied with or without a P2RX1 inhibitor (NF449), and the severity of DSS-induced mouse colitis was assessed. We observed that TNF-α mAb or NF449 treatment alone was sufficient to alleviate weight loss and DAI in DSS-treated mice (**Figures 5A, B** **)**. Moreover, weight loss and DAI were further relieved when TNF-α mAb and NF449 were used in combination. RT-qPCR results showed that expression of inflammatory cytokines was suppressed when a combination of TNF-α mAb and NF449 was used (**Figure 5C**). Next, we detected the infiltration of neutrophils in mice treated with TNF-α mAb and/or NF449. Results showed that neutrophil infiltration could be suppressed by TNF-α mAb (**Figure 5D**). Consistent with the results obtained from *P2rx1* ^−/−^ mice, NF449 could reduce neutrophil infiltration (**Figure 5D**). In mice treated with the combination of TNF-α mAb and NF449, we found that neutrophils were further inhibited when compared with the mice treated with TNF-α mAb alone. Together, these results suggest that P2RX1 antagonist can facilitate the efficiency of anti-TNF-α therapy in mouse colitis, and the mechanism is associated with decreased inflammatory cytokine and neutrophil infiltration.

## Discussion

Purinergic receptors are implicated in the pathogenesis of inflammatory disorders and are being explored as potential therapeutic targets (28). Among the P2X and P2Y receptors, P2RX7 is the most studied in the induction of inflammatory responses and has been reported to participate in the development of colitis (29, 30). Consistent with P2RX7, P2RX1 also belongs to the P2X purinergic receptor family and shared similar properties of ATP binding and ion permeability, except that P2RX1 is unable to form large pores for cytokine release (9). Published reports have identified the roles of P2RX1 in male infertility (14), vasoconstriction (31), and thrombosis (32). In addition, emerging evidence from our group (10) and others (33) further uncovers that P2RX1 is crucial for reinforcing innate and adoptive immune responses.

In the present study, by analyzing the public GEO database, we found that P2RX1 expression was significantly higher in inflamed colon of UC patients and DSS-exposed mice. Severe colitis patients are susceptible to systematic platelet dysfunction and microvascular thrombosis. Given the important role of P2RX1 in thrombosis, a recent study showed that P2RX1 deficiency inhibited peripheral platelet activation and caused subsequent coagulation dysfunction in high DSS dosage-induced severe colitis (5% for 7 days) (34). The high dosage of DSS is lethal to experimental C57BL/C mice and sufficient to induce irreversible systematic injuries (19, 35). Therefore, to explore the unidentified effect of P2RX1 on local pathological conditions, a commonly used moderate DSS dosage (2% for 7 days) was adopted to induce local intestinal inflammation. Interestingly, RNA sequencing results showed that inflammation-associated pathways as well as complement and coagulation cascade pathways were accomplished in *P2rx1* ^−/−^ mice, indicating that P2RX1 deficiency might also mediate inflammatory responses in the progression of IBD.

Etiological studies on IBD have centered on several factors, including host genetics, dysregulated immune responses, and disordered gut microbiota (2, 3). Recently, accumulating evidence has revealed the essential role of gut microbial dysbiosis in the pathogenesis of IBD (36). To explore the potential link between P2RX1 and intestinal microbiota in colitis, we performed 16S rDNA sequencing. Our results showed that basal intestinal microbiota composition was similar between WT and *P2rx1* ^−/−^ mice. However, at the acute phase (day 7) of DSS-induced colitis, major differences of intestinal microbiota composition were discovered. It suggested that host-expressed P2RX1 might contribute to shaping the plasticity of intestinal microbiota in colitis. IL-22 is characterized with the ability to induce the mucosal healing and represents potential therapeutic goal of IBD (37). Microbiota metabolites (mainly indole alkaloid)-involved AhR/IL-22 axis is important for the generation of IL-22 (15). KEGG analysis revealed that indole alkaloid biogenesis pathway was most upregulated in microbiota of *P2rx1* ^−/−^ mice. Further studies showed that IAA, indole, and subsequent IL-22 were all increased in *P2rx1* ^−/−^ mice, indicating that microbiota metabolites-involved AhR/IL-22 axis might contribute to the beneficial effects of P2RX1 ablation.

Improving the therapeutic efficacy of anti-TNF-α therapy is in urgent need for clinical treatment (7). CD is mainly driven by T helper 1 (Th1) cell-induced inflammation (with relatively high level of TNF-α), whereas UC is generally considered to be a T helper 2 (Th2) cell-mediated condition (with relatively low level of TNF-α) (38). However, the strict polarization model of Th1 and Th2 is not fully applicable in IBD due to a redundancy of effector and regulatory pathways affected by factors, such as the phase of the disease, innate inflammatory mechanisms, or anti-inflammatory treatment of patients (39). Interestingly, we found that P2RX1 expression is significantly higher in infliximab no-response UC patients, but not CD patients, which indicated that P2RX1 might provide a potential target to intervene inflammation of UC. To study whether P2RX1 inhibition could synergize with anti-TNF-α therapy, a P2RX1 inhibitor was jointly used with an anti-TNF-α mAb in DSS-induced mouse colitis. Interestingly, our results showed that the P2RX1 inhibitor significantly improved the therapeutic effects of anti-TNF-α mAb in DSS-exposed mice. From a clinical viewpoint, our study suggests a therapeutic potential of combination medication strategy for UC.

It is worth noting that our studies cannot draw a direct causal connection of the gut microbial dysbiosis and P2RX1 activation. According to previous reports, P2RX1 can facilitate the inflammatory activation of immune cells (10, 22). Our studies also showed that P2RX1 was highly expressed in the neutrophils that infiltrated inflamed intestinal tissues. Genetic knockout or pharmacologic inhibition of P2RX1 could suppress neutrophil infiltration in the acute phase of mice UC. Given that bacterial differences can also result from the gut inflammation (40), the inhibition on of neutrophil infiltration may alter the composition of gut bacteria. We suppose that antagonism of P2RX1 may have a synergetic effect of modulating microbial dysbiosis and inhibiting neutrophil infiltration in mice UC.

Together, our study reveals that P2RX1 is increased in IBD and fosters intestinal microbiota-associated inflammatory responses. Targeting P2RX1 may help to improve the anti-TNF-α therapy in IBD. Nevertheless, some unidentified issues still exist, including the specific cell contribution of P2RX1 and detailed mechanism that P2RX1 engaged to shape intestinal microbiota, which still need further work.

## Conclusions

By screening public GEO datasets, we found purinergic receptor P2RX1 was upregulated in inflamed colon tissues, and correlated with anti-TNF-α efficiency. Genetic ablation of P2RX1 relieved dextran sulfate sodium (DSS)-induced mice colitis. A P2RX1 inhibitor increased therapeutic efficiency of anti-TNF-α therapy. Mechanistically, reduced neutrophil infiltration and microbiota metabolite-involved aryl hydrocarbon receptor (AhR)/IL-22 axis are associated with the beneficial effects of P2RX1 suppression. Together, our work suggests that P2RX1 may be a potential therapeutic target of IBD.

## Data Availability Statement

Sequencing data have been deposited in the Sequence Read Archive (SRA) repository under accession number PRJNA658839 (https://www.ncbi.nlm.nih.gov/sra/?term=PRJNA658839).

## Ethics Statement

All experimental protocols were approved by the Council on Animal Care at Jiangsu University on the Protection and the Welfare of Animals and followed the National Institutes of Health of China guidelines for the care and use of experimental animals.

## Author Contributions

XY, YS, and JH performed animal experiments. QJ and SF analyzed the GEO database. RL and LH analyzed the RNA-sequencing data. XW and CD designed the study and wrote the article. All authors contributed to the article and approved the submitted version.

## Funding

This study was supported by the National Natural Science Foundation of China (ID: 81701945 to XW); China Postdoctoral Science Foundation (ID 2018M640403 to XW).

## Conflict of Interest

The authors declare that the research was conducted in the absence of any commercial or financial relationships that could be construed as a potential conflict of interest.

## Supplementary Material

Supplementary Figure 1 Pathological examinations of DSS-exposed WT and P2rx1^−/−^ mice. WT and *P2rx1* ^−/−^ mice were treated with 2% DSS for 7 days. Intestinal tissues from D0 and D7 mice were harvested and H&E staining was performed (scale bar 1 mm for high power fields, 50 μm for low power fields).

Supplementary Figure 2 Expression pattern of P2RX1 in inessential tissue. Expression pattern of P2RX1 in inessential tissue was scanned in public HPA single cell sequencing database (https://www.proteinatlas.org/).

Supplementary Figure 3 Flow cytometry analyses of neutrophil and macrophage infiltration in DSS-exposed WT and P2rx1^−/−^ mice. WT and *P2rx1* **^−^** ^/^ **^−^** mice were treated with 2% DSS for 7 days. Ly6G was used to label neutrophils, and F4/80 was used to label macrophages. Infiltrated neutrophils and macrophages and P2RX1 expression in each cell type were determined at D0 and D7.

Supplementary Figure 4 Composition of intestinal microbiota species in DSS-exposed WT and *P2rx1* ^−/−^ mice. WT and *P2rx1* **^−^** ^/^ **^−^** mice were treated with 2% DSS for 7 days. At days 0, 7, and 12, fecal microbiota was quantified using 16S rDNA sequencing. Detailed composition of intestinal microbiota species in each group was shown (n = 6 for D0 WT and *P2rx1* **^−^** ^/^ **^−^** groups, n = 3 for the rest groups).

Supplementary Figure 5 Composition of intestinal microbiota species in DSS-exposed WT and *P2rx1* ^−/−^ mice. WT and *P2rx1* **^−^** ^/^ **^−^** mice were treated with 2% DSS for 7 days. At day 7, fecal microbiota was quantified using 16S rDNA sequencing. Detailed composition of intestinal microbiota species in each group was shown (n = 3 for each group).

Supplementary Figure 6 Expression of AhR target genes in DSS-exposed WT and *P2rx1* ^−/−^ mice. WT and *P2rx1* **^−^** ^/^ **^−^** mice were treated with 2% DSS for 7 days. At day 7, colon tissues were harvested and two AhR target genes, *CYP1A1* and *CYP1B1*, were detected by RT-qPCR (n = 4 per group).

Supplementary Figure 7 Genotyping results of WT and P2rx1-/- mice. PCR was performed for genotyping gene-edited mice. As shown in the representative image, No. 370, 374 and 378 were WT mice, whereas No. 372 and 376 were P2rx1-/- mice. Heterozygous parent (P), C57BL/6 (B6) and null (N) were set as controls. The rest are heterozygous mice.
