# Supplementary material for: Targeting Purinergic Receptor P2RX1 Modulates Intestinal Microbiota and Alleviates Inflammation in Colitis
Source: Front Immunol. 2021 Jul 20;12:696766. doi: 10.3389/fimmu.2021.696766 (PMC8329583; doi:10.3389/fimmu.2021.696766)
Supplement: Supplementary Figure 1 — Pathological examinations of DSS-exposed WT and P2rx1−/− mice. WT and P2rx1 −/− mice were treated with 2% DSS for 7 days. Intestinal tissues from D0 and D7 mice were harvested and H&E staining was performed (scale bar 1 mm for high power fields, 50 μm for low power fields). [file DataSheet_1.docx]

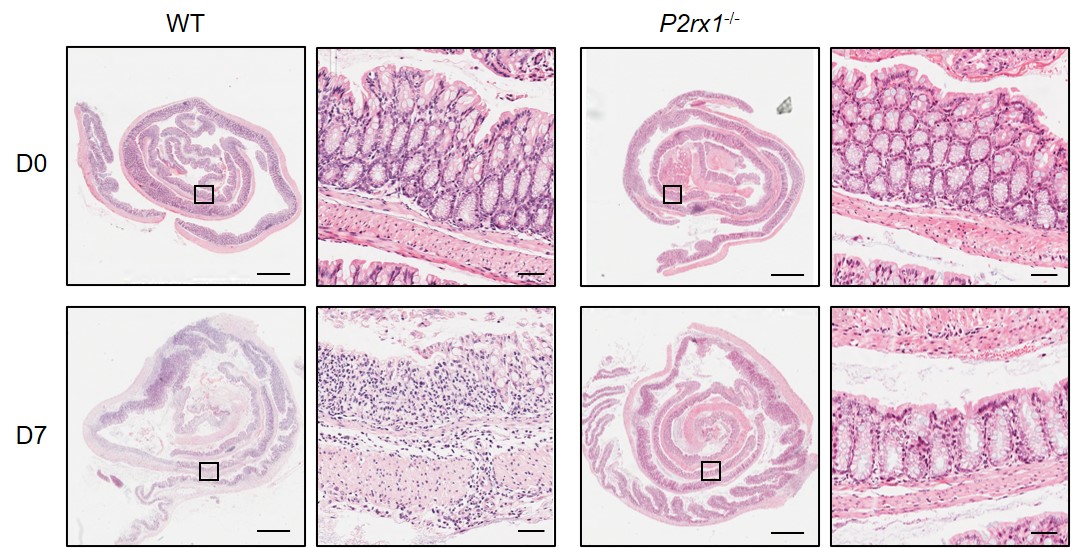


**Figure S1. Pathological examinations of DSS-exposed WT and P2rx1-/- mice.** WT and *P2rx1*^-/-^ mice were treated with 2% DSS for 7 days. Intestinal tissues from D0 and D7 mice were harvested and H&E staining was performed (Scale bar 1mm for high power fields, 50μm for low power fields).
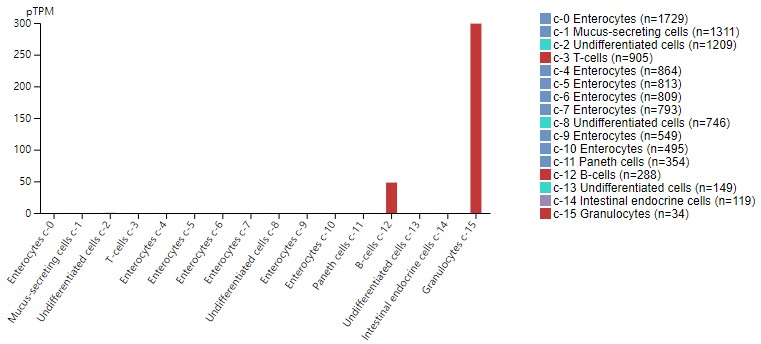


**Figure S2. Expression pattern of P2RX1 in inessential tissue.** Expression pattern of P2RX1 in inessential tissue was scanned in public HPA single cell sequencing database (https://www.proteinatlas.org/).


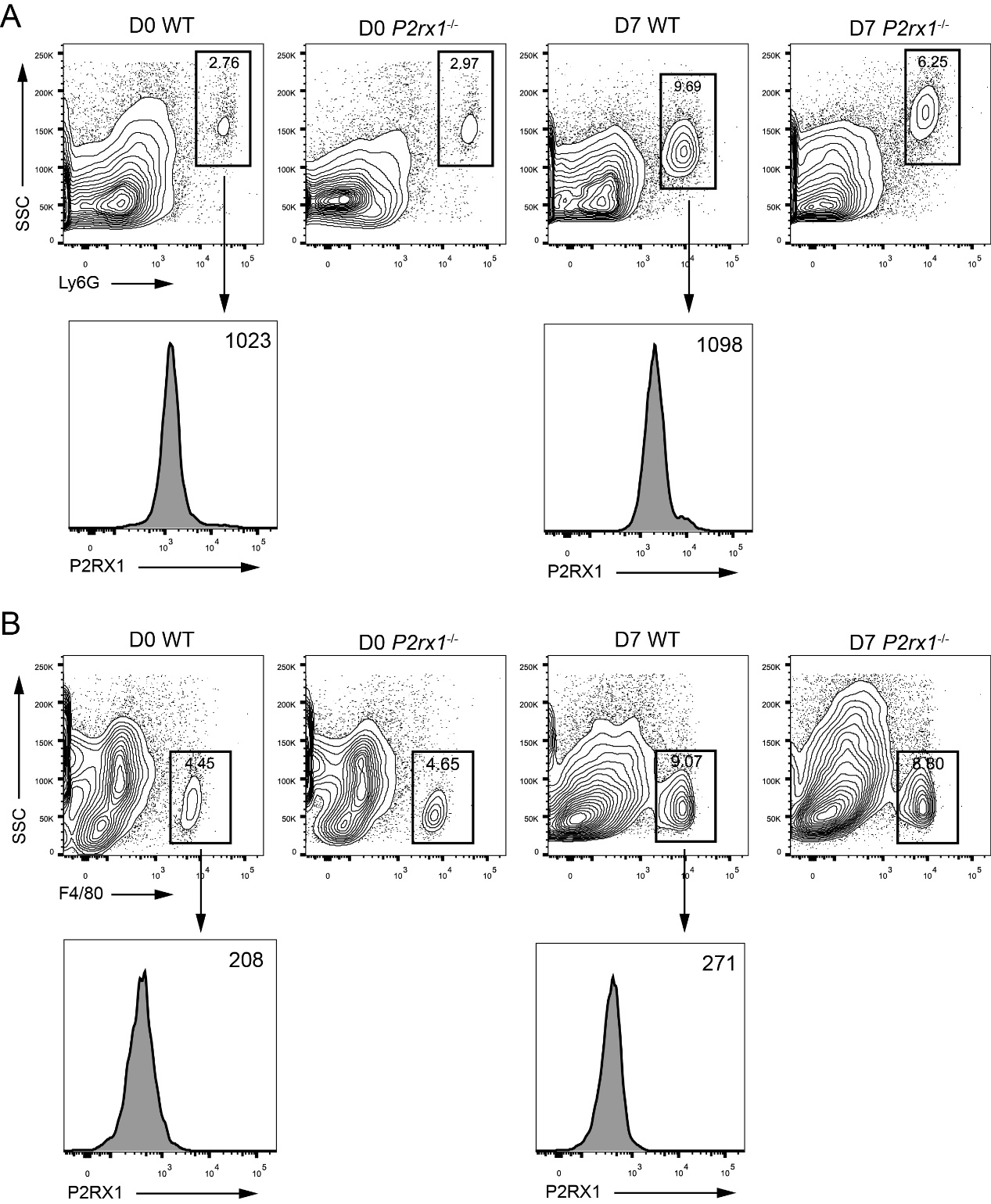


**Figure S3. Flow cytometry analyses of neutrophils and macrophages infiltration in DSS-exposed WT and P2rx1-/- mice.** WT and *P2rx1*^-/-^ mice were treated with 2% DSS for 7 days. Ly6G was used to label neutrophils, and F4/80 was used to label macrophages. Infiltrated neutrophils and macrophages, and P2RX1 expression in each cell type were determined at D0 and D7.


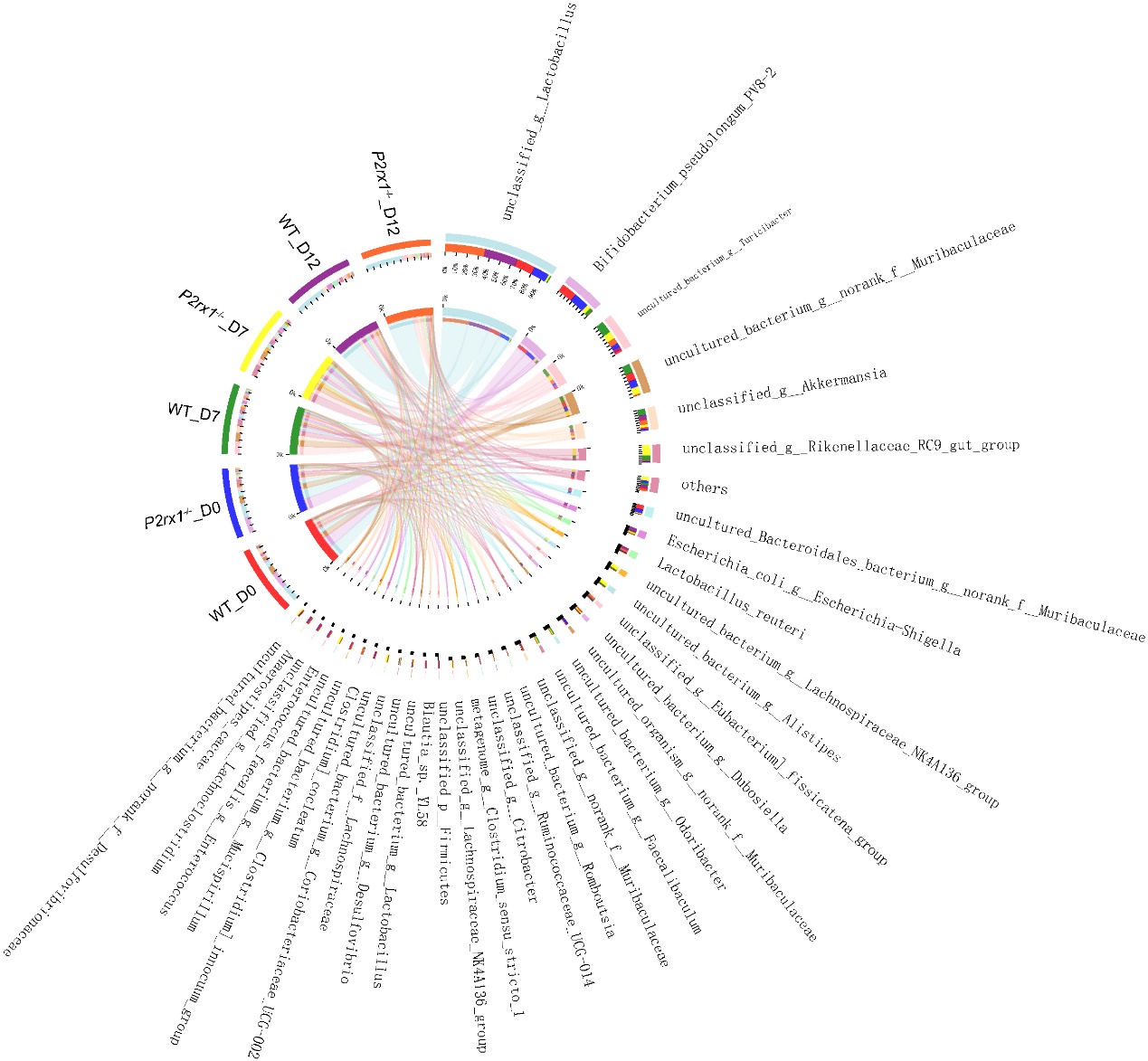


**Figure S4. Composition of intestinal microbiota species in DSS-exposed WT and *P2rx1*^-/-^ mice.** WT and *P2rx1*^-/-^ mice were treated with 2% DSS for 7 days. At day 0, 7 and 12, fecal microbiota was quantified using 16S rDNA sequencing. Detailed composition of intestinal microbiota species in each group was shown (n=6 for D0 WT and *P2rx1*^-/-^ groups, n=3 for the rest groups).


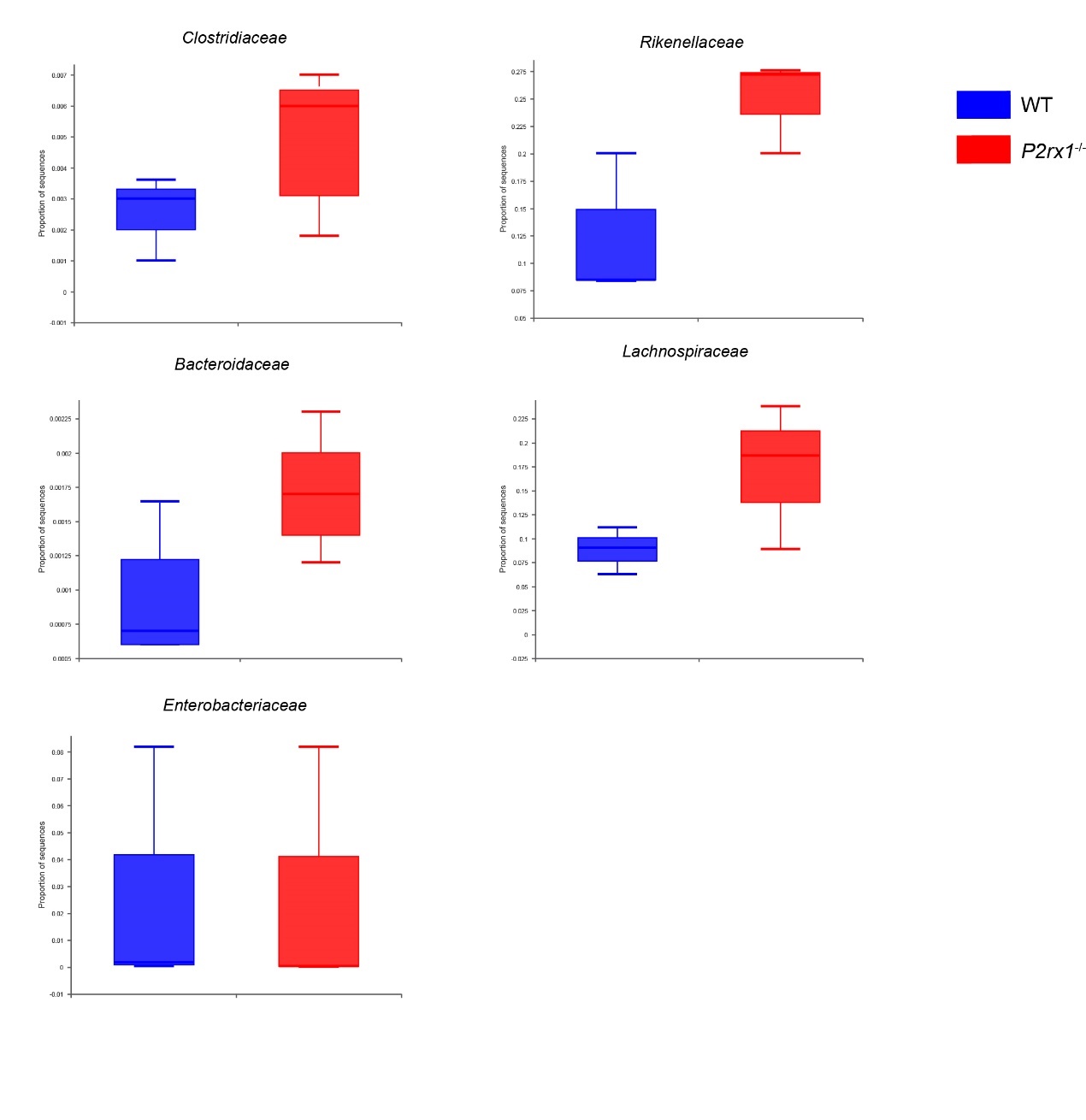


**Figure S5. Composition of intestinal microbiota species in DSS-exposed WT and *P2rx1*^-/-^ mice.** WT and *P2rx1*^-/-^ mice were treated with 2% DSS for 7 days. At day 7, fecal microbiota was quantified using 16S rDNA sequencing. Detailed composition of intestinal microbiota species in each group was shown (n=3 for each group).


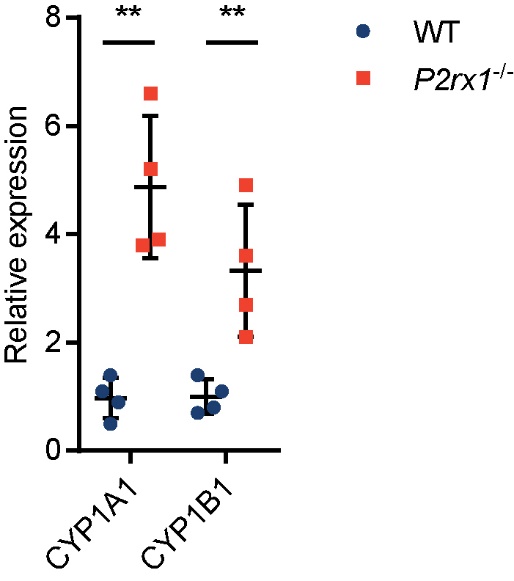


**Figure S6. Expression of AhR target genes in DSS-exposed WT and *P2rx1*^-/-^ mice.** WT and *P2rx1*^-/-^ mice were treated with 2% DSS for 7 days. At day 7, colon tissues were harvested and two AhR target genes, CYP1A1 and CYP1B1, were detected by RT-qPCR (n=4 per group).


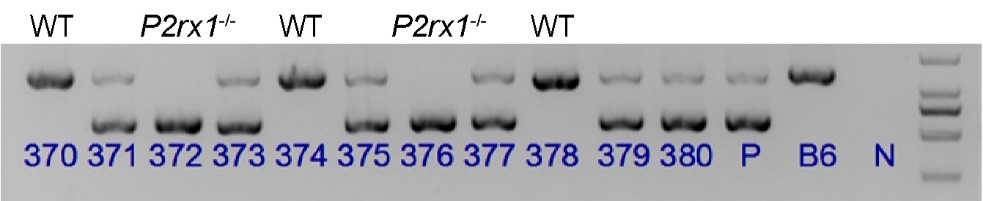


**Figure S7. Genotyping results of WT and *P2rx1*^-/-^ mice.** PCR was performed for genotyping gene-edited mice. As shown in the representative image, No. 370, 374 and 378 were WT mice, whereas No. 372 and 376 were *P2rx1*^-/-^ mice. Heterozygous parent (P), C57BL/6 (B6) and null (N) were set as controls. The rest are heterozygous mice.
